# Whole genome sequencing reveals mycobacterial microevolution among concurrent isolates from sputum and blood in HIV infected TB patients

**DOI:** 10.1186/s12879-016-1737-2

**Published:** 2016-08-05

**Authors:** Willy Ssengooba, Bouke C. de Jong, Moses L. Joloba, Frank G. Cobelens, Conor J. Meehan

**Affiliations:** 1 Department of Medical Microbiology, College of Health Sciences Makerere University, Kampala, Uganda; 2 Unit of Mycobacteriology, Institute of Tropical Medicine, Antwerp, Belgium; 3 Department of Global Health and Amsterdam, Institute of Global Health and Development, Academic Medical Center, University of Amsterdam, Amsterdam, Netherlands; 4 KNCV Tuberculosis Foundation, The Hague, Netherlands; 5 Division of Infectious Diseases, New York University, New York, NY USA

**Keywords:** Intra-patient, Microevolution, Ancestral, Concurrent sputum and blood *M. tuberculosis*

## Abstract

**Background:**

In the context of advanced immunosuppression, *M. tuberculosis* is known to cause detectable mycobacteremia. However, little is known about the intra-patient mycobacterial microevolution and the direction of seeding between the sputum and blood compartments.

**Methods:**

From a diagnostic study of HIV-infected TB patients, 51 pairs of concurrent blood and sputum *M. tuberculosis* isolates from the same patient were available. In a previous analysis, we identified a subset with genotypic concordance, based on spoligotyping and 24 locus MIRU-VNTR. These paired isolates with identical genotypes were analyzed by whole genome sequencing and phylogenetic analysis.

**Results:**

Of the 25 concordant pairs (49 % of the 51 paired isolates), 15 (60 %) remained viable for extraction of high quality DNA for whole genome sequencing. Two patient pairs were excluded due to poor quality sequence reads. The median CD4 cell count was 32 (IQR; 16–101)/mm^3^ and ten (77 %) patients were on ART. No drug resistance mutations were identified in any of the sequences analyzed. Three (23.1 %) of 13 patients had SNPs separating paired isolates from blood and sputum compartments, indicating evidence of microevolution.

Using a phylogenetic approach to identify the ancestral compartment, in two (15 %) patients the blood isolate was ancestral to the sputum isolate, in one (8 %) it was the opposite, and ten (77 %) of the pairs were identical.

**Conclusions:**

Among HIV-infected patients with poor cellular immunity, infection with multiple strains of *M. tuberculosis* was found in half of the patients. In those patients with identical strains, whole genome sequencing indicated that *M. tuberculosis* intra-patient microevolution does occur in a few patients, yet did not reveal a consistent direction of spread between sputum and blood. This suggests that these compartments are highly connected and potentially seed each other repeatedly.

**Electronic supplementary material:**

The online version of this article (doi:10.1186/s12879-016-1737-2) contains supplementary material, which is available to authorized users.

## Background

The recent advances in molecular analytical methods have increased our understanding of the possible heterogeneity of infection with *Mycobacterium tuberculosis* [1]. Several perspectives around this complexity in relation to HIV-infection have been documented [2]. However, little is known on the intra-patient mycobacterial diversity and direction of seeding between the sputum and blood compartments.

Clonal variants can be detected using Variable Number of Tandem Repeats (VNTR) [3, 4] or Restriction Fragment Length polymorphism (RFLP) of the IS6110-typing genetic elements [5, 6]. The subtle genetic rearrangements caused by microevolution in IS6110 [7] are known to interrupt genes or modulate the expression of adjacent genes. These can affect interpretation of molecular epidemiological tests [8–10], whereas if this happens in the VNTR regions, such changes can modify the transcription of neighboring genes [11, 12]. These changes may have a role in the infectivity of the bacteria [13] and their survival within the host [14–17]. In a published report, it was suggested that such microevolution affected cavity formation, hence increased transmissibility of the emerging clonal variants [17].

The advent of whole genome sequencing (WGS) has led to the identification of several limitations of traditional molecular epidemiological methods in ascertaining microevolution occurring outside the classical targeted genetic elements [18, 19]. Micro-evolutionary changes may further be modified against a background of impaired immunity as a result of AIDS. Whether HIV/AIDS is the main cause of a systematic heterogeneity of a within-host population of *M. tuberculosis* as a result of advanced immune suppression [20] or as a result of pathogen microevolution, remains a challenge. A recent study involving four patients found as high a genetic diversity within as between patients [21].

In the present study, we considered a large cohort of HIV-positive patients who had concurrent pulmonary and blood *M. tuberculosis* strains and were categorized as identical, up to one spacer and/or locus difference, using the conventional methods of spoligotyping (spacer oligonucleotide typing) and Mycobacterial Interspersed Repetitive Units (MIRU)-VNTR 24 loci. We applied whole genome sequencing (WGS) to study microevolution among these strains by documenting differences in distribution of single-nucleotide polymorphisms (SNPs) between strains isolated from sputum and blood. We also aimed at ascertaining the ancestral *M. tuberculosis* strain between sputum and blood in each patient.

## Methods

### Study participants

From a previous study of 51 HIV-infected TB patients who had concurrent blood and sputum *M. tuberculosis* isolates at enrolment, we selected patients found to have identical genotypes from our previous study [22] using both spoligotyping [5] and MIRU-VNTR 24 loci methods [3]. In this strain selection process, we considered pairs (*n* = 25) with maximum one spacer and/or locus difference to be identical MTB-genotypes.

### DNA sequencing

Whole genome sequencing of the DNA from *M. tuberculosis* isolates was performed on an Illumina HiSeq platform at Genoscreen (Lille, France) or the Beijing Genome Institute (BGI), China following the Illumina TruSeq DNA sample preparation recommendations.

### SNP and indel calling for genotype and drug resistance

To confirm genotypic classification assigned using previous methods, sequences were processed through an online program, PhyResSE, which assigns lineages after calling SNP and indels that are known to be lineage specific [23, 24]. Since dynamic changes in *M. tuberculosis* have been found to occur during acquisition and fixation for drug resistance [25], we also used the same program to call for drug-specific SNPs and indels.

### Mapping of the fastq reads and complete variant calling

For each sequence, we used the nesoni version 0.13 pipeline (https://github.com/Victorian-Bioinformatics-Consortium/nesoni) to remove Illumina adaptors sequences and low quality bases from reads using a minimum read quality of 10 and length of 45. We employed the nesoni bowtie tool for read alignment using the most recent common ancestor of the *M. tuberculosis* complex (MTBc; H37rv_NC_018143.1) as referenced [26, 27]. To look for differences between the reads and the reference genome, we used nesoni consensus to process the mapped reads for the SNP calling process. Quality mapping thresholds included removing reads that mapped to more than 1 position, minimum coverage of 10, minimum mapping quality of the SNP of 20 and minimum read coverage of 66 %.

A tabular list of all SNPs and indels per isolate was created using nesoni nway (Additional file 1), from which a SNP alignment was created using custom python scripts.

### Phylogenetic analysis of sequence data

To infer intraspecific phylogenies with the expected small distances, we constructed a maximum likelihood tree using Randomized Axelerated Maximum Likelihood (RAxML) version 8.2 [28], based upon the SNP alignment and employing a generalized time-reversible (GTR) CAT model with Stamatakis ascertainment bias correction [28]. We calculated 100 bootstrap replicates for support of the tree nodes. We also created a neighbor joining distance matrix based upon the SNP alignment using the Molecular Evolutionary Genetics Analysis (MEGA) version 5.2.2 [29].

## Results

**Fig. 1:**
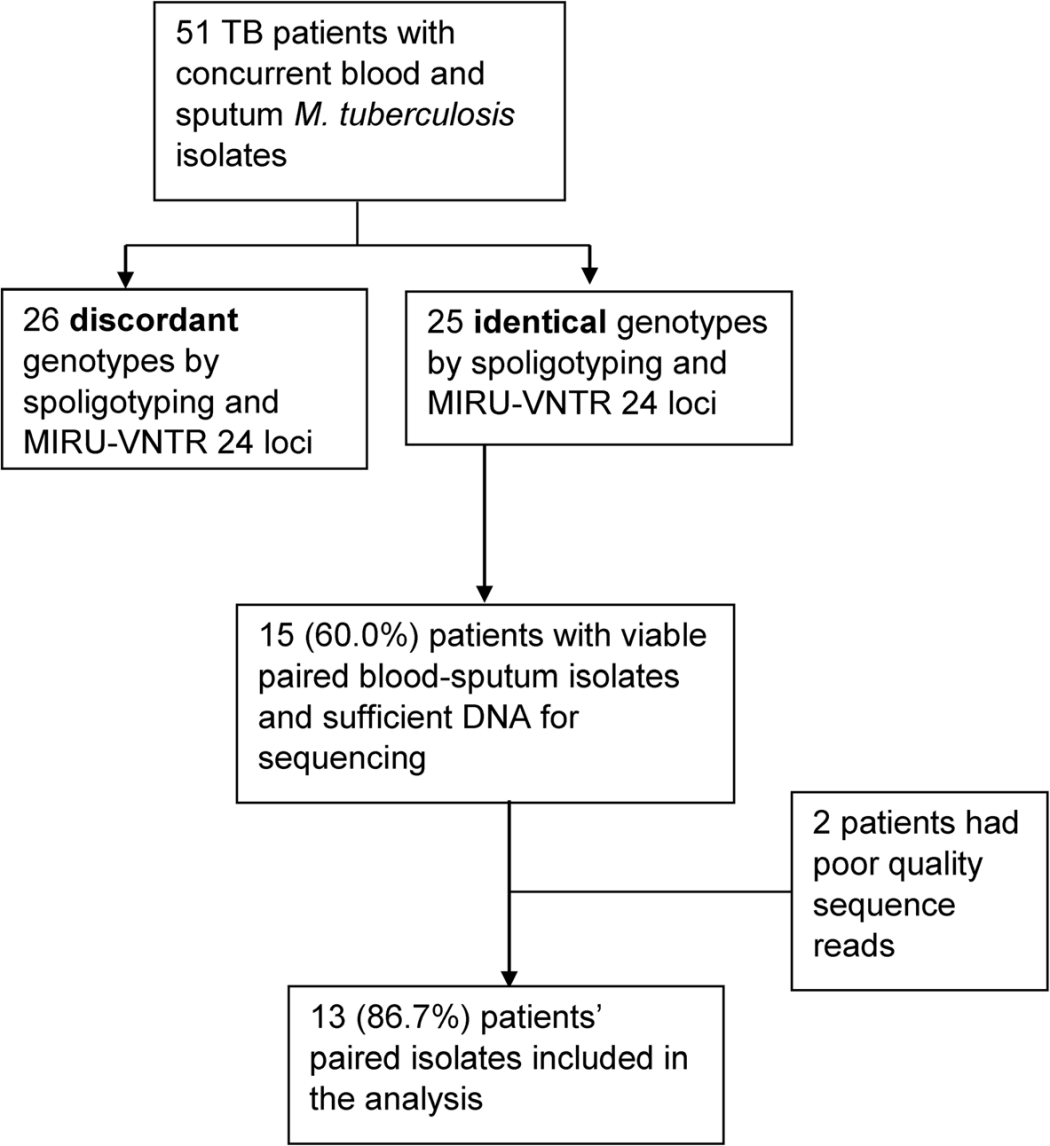
Flow chart showing the participant with concurrent sputum and blood *M. tuberculosis* sequences. MIRU = Mycobacterial Interspersed Repetitive Units, VNTR = Variable Number of Tandem Repeats

Of the 25 patients with identical *M. tuberculosis* genotypes, 15 (60.0 %) had viable mycobacterial bacilli on subculture and enough DNA for whole genome sequencing. Two isolates had sequence reads of poor quality and were eliminated from the analysis. We analyzed data for 13 (86.7 %) of the patients with good quality sequence reads from both blood and sputum (Fig. 1).

**Fig. 2:**
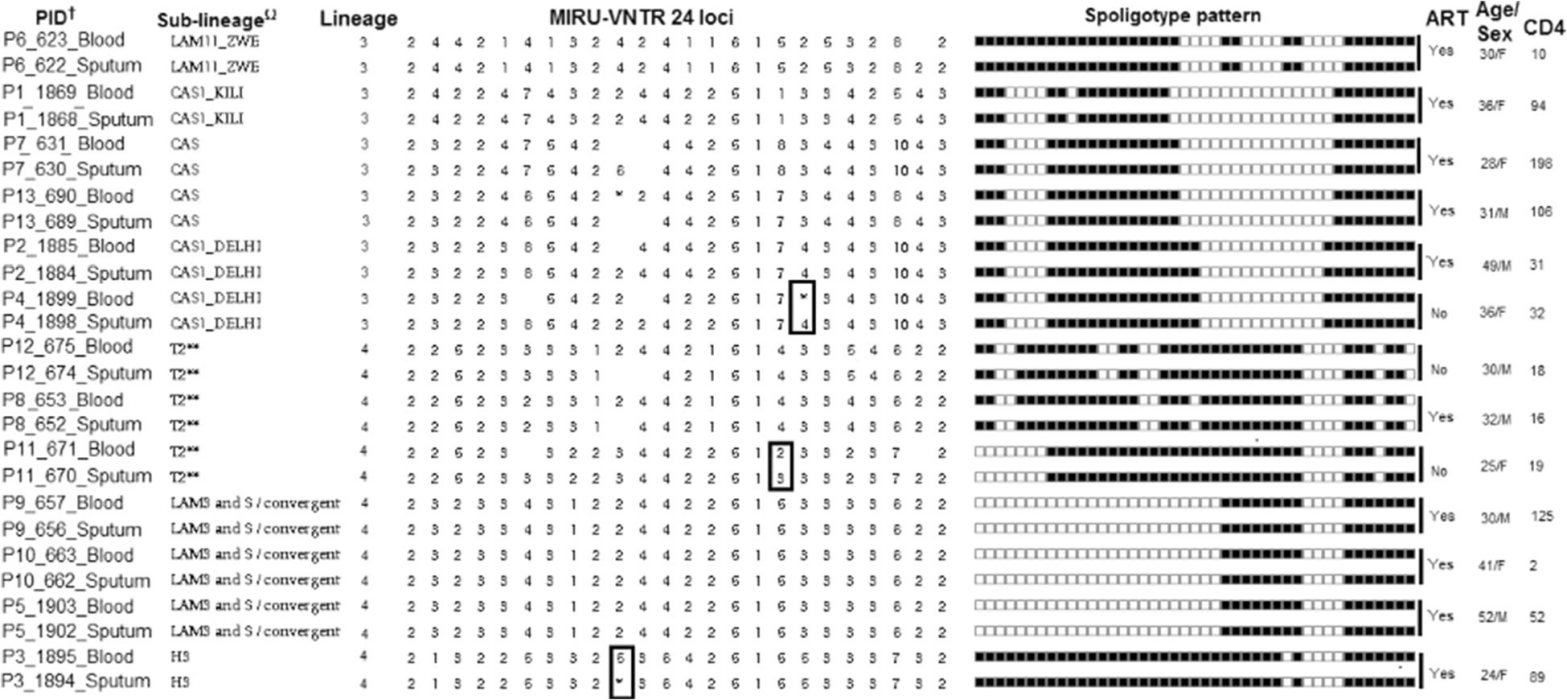
Participants’ with concurrent sputum and blood *M tuberculosis* isolates and DNA sequences. Ω = lineage based on spolDB4 online database, sub-lineage ** = spolDB4 unassigned with T2 SNPs, MIRU-VNTR = Mycobacterial Interspersed Repetitive Unit-Variable-Number Tandem Repeat, MIRU-VNTR* = Mixed allele at that locus, † = All drug susceptible and with no resistance mutations, Squared pairs = single locus variants, Rectangle in MIRU table = single locus variants (SLV)

The 13 patients included 8 (61.5 %) women and had a median age of 32 (interquartile range; IQR, 28–37) years. The median CD4 cell count was 32 (IQR; 16–101)/mm^3^; 10 (76.9 %) were taking ART and only one patient was previously treated for tuberculosis. Lineage assignments based on SNP detection found five (38.5 %) patients had *M tuberculosis* lineage three (L3; Delhi/CAS) whereas eight (61.5 %) had lineage four (L4; Haarlem and LAM11_ZWE each 12.5 %, LAM3 and S convergent and T2; each 37.5 %) (Fig. 2). These results were in line with those found by spoligotype in the parent study [22]. The SpolDB4 unassigned T2 were found to have SNPs specific to T2- Uganda including mutations in the gyrA gene at position T80A. No drug resistance mutations were identified in any of the sequences analyzed (Fig. 2).

### SNP calling and neighbor joining tree for concurrent sputum and blood *M tuberculosis* isolates

Of the 13 patients, three (23.1 %) had SNPs (indicating evidence of microevolution) detected when comparing their concurrent sputum and blood *M. tuberculosis* isolates. One SNP was seen in a patient’s pair that was considered a clonal variant, single locus variants (SLV) by MIRU-VNTR 24 loci (Fig. 2) and the two were from identical pairs between pulmonary and blood compartments. The identified SNPs, their corresponding H_37_RV genome coordinates and the gene function as stated in Tuberculist online database [30] are indicated (Additional file 2).

**Fig. 3:**
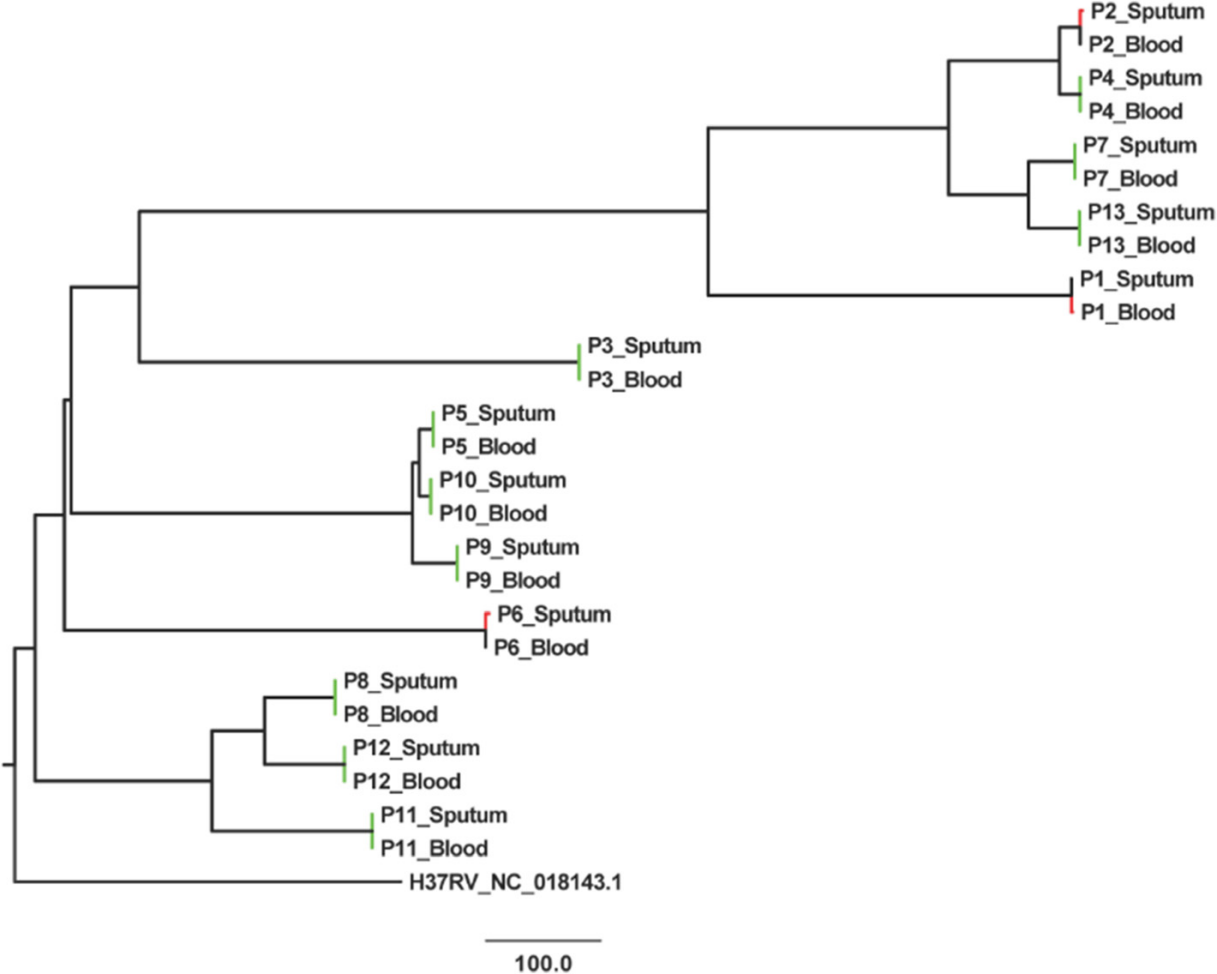
Neighbor Joining SNP distance matrix based tree for potential ancestral strain comparing sputum and blood *M. tuberculosis* strains. Created using MEGA 5.2.2; Visualized and colored using Fig Tree 1.4.2 (http://tree.bio.ed.ac.uk/software/figtree/); Black = Ancestral strain, Green = Identical strains, p = patient, Red = descendant strains

The RAxML analysis using SNPs did not show a difference in branch lengths and thus could not determine the ancestral strain between most of blood vs. sputum pairs. We therefore performed phylogenetic analysis to identify the ancestral compartments through a distance matrix derived NJ tree generated using MEGA. In a total of two (15.4 %) patients the blood isolate was ancestral to the sputum isolate, in one (7.7 %) it was the opposite, and ten (76.9 %) of the pairs were identical (Fig. 3).

## Discussion

The advent and extended use of WGS strategies have increased our understanding of the transmission, epidemiological and molecular dynamics of the *M. tuberculosis* pathogen [1, 18, 31]. Recently, WGS analysis has been mainly applied to identify the number of SNPs to document the *M. tuberculosis* micro-evolutionary events between and within patients [21, 32]. Intra-patient *M. tuberculosis* microevolution has been found to be similar to the inter-patient microevolution and has been suggested to impact on the expected strain diversity within a transmission chain [21].

In the current study, we applied WGS to isolates of *M. tuberculosis*, which were identical by conventional typing methods, from HIV-infected patients with poor cellular immunity, in half of whom infection with multiple strains of *M. tuberculosis* was found. Our study documented three (23.1 %) patients with SNPs (indicating evidence of microevolution) when comparing their concurrent sputum and blood *M. tuberculosis* isolates. Although high intra-patient variability may be expected during the process of resistance acquisition [25, 33], all isolates in our study from both compartments were drug susceptible with no resistance conferring mutations. Categorizing these isolates as identical by both spoligotyping and MIRU-VNTR 24 loci typing methods, yet with different SNPs, underscores the power of whole genome sequencing in ascertaining microevolution occurring outside the classical targeted genetic elements of *M. tuberculosis* compared to traditional molecular epidemiological methods [18, 19]*.* Small changes have been implicated to influence bacterial phenotypes, such as strain infectivity [13] and within-host pathogen survival [14–17].Moreover, clonal MDR-variants of concurrent pulmonary and disseminated tuberculosis strains have been documented [34] which need to be recognized for appropriate therapy to be initiated. More complex intra-patient microevolution of MDR-MTBC strains under treatment has been documented through WGS analysis [35].

Studies have suggested dissemination of pulmonary tuberculosis is due to impaired immunity including compartmentalization [2, 20, 36, 37] and/or reinfection [38]. Some studies have hypothesized pulmonary infection as a spill-over of the lymphatic or haematogenous dissemination of tuberculosis [39–41]. However, few studies have approached these hypotheses using concurrent clinical *M. tuberculosis* isolates. Through a neighbor joining SNP distance matrix based tree, the present study found *M. tuberculosis* cross-seeding between pulmonary and blood compartments using clinical *M. tuberculosis* isolates. This may be due to the high connectedness of these compartments that may lead to repeated seeding in-between these compartments under extensive immunosuppression. Blood as the origin of tuberculosis disease, contrary to the dogma, may be supported by the fact that *M. tuberculosis* can persist in several sites and cell types that might constitute reservoirs that can reactivate infection producing extrapulmonary tuberculosis with or without lung involvement [42]. Indeed in the main study, 12/182 (6.6 %) of the tuberculosis patients had MTB cultured from blood with two negative sputum cultures [43]. The strains with no clear direction of seeding between sputum and blood compartments could have been influenced by advanced HIV/AIDS immune suppression resulting to increased early dissemination [20].

Our study has some limitations; complete genome sequences can currently only be obtained from cultured isolates, which may have introduced a bias, as mixed infections may have been missed if culture favored one genotype [44]. However, where blood and sputum MTB strains were identical, this strongly suggests blood and sputum acted as one compartment. Conversely, these findings validate sequencing techniques and suggest that in vitro culture did not add significant bias. Furthermore, we cannot exclude that microevolution occurred in vitro and also could not compare the observed micro-evolutionary changes between different levels of impaired immunity. However, the current study suggests that *M. tuberculosis* micro-evolutionary events can occur over a short time scale during disease progression.

Additionally, although the small sample size of our study may have reduced the power of our conclusions, it is worth noting that the isolation of paired strains from blood and sputum is notoriously difficult. This is due to the fact that mycobacteremia only occurs in patients with advanced immunosuppression, who fortunately are less prevalent since the wide roll-out of antiretroviral therapy. Moreover, since the most widely used automated liquid culture system today, the BD MGIT 960, is not designed for mycobacterial blood cultures, we expect that our sample size will unlikely be surpassed by future studies.

## Conclusions

In conclusion, among HIV-infected patients with poor cellular immunity, infection with multiple strains of *M. tuberculosis* was found in half of the patients. In the patients with identical strains, whole genome sequencing showed minimal M*. tuberculosis* intra-patient microevolution and did not reveal a consistent direction of spread between sputum and blood, suggesting that these compartments are highly connected and potentially seed each other repeatedly. However, SNP analysis of the whole genome sequencing results indicates that micro-evolutionary events can occur even over a short time scale during disease progression, and may be observed even in a small samples size. Future studies are needed to enrich our understanding of the role of microevolution in tuberculosis disease presentation and progression. The almost binary distinction between infections with different strains versus the apomictic identical strains that populate blood and sputum compartments warrants further investigation. We recommend a larger set of sputum/blood pairs to support the interpretation that microevolution occurs and that reseeding continually occurs between compartments among severely immunocompromised HIV-infected individuals. This would also allow for an assessment of the clinical parameters that may be associated, for example, with viral suppression status due to, ART.

## Abbreviations

DNA, deoxy nucleic acid; GTR, generalized time reversible; HIV, human immunodeficiency virus; MDR-TB, multi-drug resistant tuberculosis; MEGA, molecular evolutionary genetics analysis; MIRU, mycobacterial interspersed repetitive unit; MTB, mycobacterium tuberculosis; RAxML, randomized axelerated maximum likelihood; RFLP, restriction fragment length polymorphism; SLV, single locus variant; SNP, single nucleotide polymorphisim; VNTR, variable number of tandem repeats; WGS, whole genome sequencing

## Additional files

Additional file 1: A tabular list of all SNPs and indels per M*. tuberculosis* isolate, sputum and blood, created using nesoni nway program. Key: Highlighted = SNPs between concurrent sputum and blood M. tuberculosis isolates, *p* = patient. (XLS 15043 kb) Additional file 2: SNPs and SNP functions identified by comparing concurrent sputum and blood *M. tuberculosis* strains from HIV-infected individuals. (DOC 30 kb)
