# Supplementary material for: Whole genome sequencing reveals mycobacterial microevolution among concurrent isolates from sputum and blood in HIV infected TB patients
Source: BMC Infect Dis. 2016 Aug 5;16:371. doi: 10.1186/s12879-016-1737-2 (PMC4974755; doi:10.1186/s12879-016-1737-2)
Supplement: Additional file 2: — SNPs and SNP functions identified by comparing concurrent sputum and blood M. tuberculosis strains from HIV-infected individuals. (DOC 30 kb) [file 12879_2016_1737_MOESM2_ESM.doc]

| **PID** | **Position in H_37_RV** | **Gene Name** | **Function (Tuberculist)** | **Sputum /Blood** |
| --- | --- | --- | --- | --- |
| P1_1868/1869 | 4065143 | Rv3626c | Function unknown; conserved hypotheticals | C=>T |
| P2_1884/1885 | 2171602 | unknown | Synonymous | T=>C |
|  | 3204327 | unknown | Synonymous | G=>T |
|  | 2779023 | Rv2476c | Catabolic glutdh involved in the utilization of glutamate and other amino acids of the glutamate family. | C=>deletion |
| P6_622/623 | 871525 | Rv0778c | Gene encodes for Cytochromes P450, a group of heme-thiolate monooxygenases. They oxidize a variety of structurally unrelated compounds, including steroids, fatty acids, and xenobiotics. | G=>A |
|  | 1319772 | Rv1181c | Potentially involved in some intermediate steps for the synthesis of a polyketide molecule which may be involved in secondary metabolism. | G=>A |
|  | 3780698 | Rv3368c | Function unknown; probably involved in cellular metabolism | A=>G |
